# The efficacy and mechanism of acupuncture in the treatment of male infertility: A literature review

**DOI:** 10.3389/fendo.2022.1009537

**Published:** 2022-10-18

**Authors:** Jiaxing Feng, Hui He, Yu Wang, Xu Zhang, Xiuying Zhang, Tiantian Zhang, Mengyi Zhu, Xiaoke Wu, Yuehui Zhang

**Affiliations:** ^1^ Heilongjiang University of Chinese Medicine, Harbin, China; ^2^ First Affiliated Hospital, Heilongjiang University of Chinese Medicine, Harbin, China; ^3^ Heilongjiang Provincial Hospital, Harbin Institute of Technology, Harbin, China

**Keywords:** male infertility, acupuncture, abnormal semen parameters, male sexual dysfunction, varicocele, genital inflammation

## Abstract

Fertility, a social, cultural, and medical issue, has aroused public attention because of its potential to predict future health. In recent years, the incidence of male infertility has increased significantly, and various risk factors, such as congenital factors, acquired factors, and idiopathic factors, have led to this situation. Male infertility causes substantial psychological and social distress in patients. With the implementation of the two-child policy, male infertility has brought enormous psychological and social pressure and huge economic burden to patients and the healthcare system. This has attracted the attention of not only men of childbearing age but also many male experts. The conventional therapeutic approaches for treating male infertility, including drugs, varicocele surgery, intrauterine insemination, *in vitro* fertilization, and intracytoplasmic sperm injection, can restore fertility to a certain extent, but their efficacy is far from satisfactory, not to mention some adverse events. Therefore, acupuncture has been chosen by many men to treat their infertility and produced significant effects. In the present paper, the efficacy and mechanism of acupuncture in the treatment of male infertility were analyzed from different perspectives such as regulating hormone secretion, reducing inflammation, and improving semen parameters. The existing literature shows that acupuncture can effectively treat male infertility.

## Introduction

Infertility is usually defined as being unable to get pregnant after unprotected intercourse for more than 12 months (1). It is estimated that approximately 15% of couples might suffer from infertility, of which the male factor accounts for nearly 50% (2, 3). Agawal et al. studied the regions and reports of male infertility rate according to the data of female infertility and reported that there are at least 30 million male infertility in the world (4). Although great advancements have been made, the etiology of more than half of male patients with infertility still cannot be determined (5).

Apart from increasing the burden on women, the lack of evaluation of male infertility often leads to neglect of several diseases. Numerous literatures suggest that there is an intimate association between male infertility and some medical conditions, including oncological, cardiovascular, autoimmune, and metabolic disorders, and even mortality (6). Several studies have illustrated that the impaired semen parameters (e.g., semen volume, sperm concentration, sperm motility, and total sperm count) have a positive correlation with the increased risk of sperm mortality (7, 8). Meanwhile, owing to not being covered by essential health insurance, the treatment of infertility imposes a considerable economic burden on patients and the healthcare system. Although the awareness of infertility has increased, its legislation covered by health insurance still needs cooperation from different apartments (9). Therefore, effective measures must be taken to avoid the above situation, especially the early diagnosis and proper management.

The treatment of male infertility needs to be formulated depending on individual etiology and need. Couples better quest for high-quality counseling to acquire proper diagnostic tests and the most appropriate therapy, including pharmacology, surgery, and assisted reproductive technology (10). However, some of the aforementioned methods are still controversial, for instance, the indications and surgical methods for varicocele repair (11). The semen parameter can be improved by varicocele repair in men with varicoceles and abnormal semen quality and quantity. However, current guidelines do not recommend varicocelectomy in infertile men with normal semen analysis or men with sub-clinical varicocele (12). In addition, these therapies also have some shortcomings, such as low live-birth rate, high cost, a long-cycle treatment, and adverse reactions. Complementary and alternative medicine (CAM) encompasses a broad set of practices including the use of acupuncture, natural products, and mind–body therapies, which is defined by the Centers for Disease Control (CDC) as a medical and healthcare system that differs from current conventional medicine (13, 14). Rayner et al. estimated that 29%–91% of fertility patients utilized a CAM method during their treatment (15). During fertility treatment, CAM could increase hope by incorporating cultural traditions of health and fertility (16). Meanwhile, it was also reported that CAM could increase the pregnancy rate and decrease stress and anxiety levels (17).

Acupuncture, a branch of traditional Chinese medicine (TCM), is a therapy that combines meridians, acupuncture points, and acupuncture methods to treat diseases for thousands of years in China (18). TCM believes that there is a network called meridians in the body to connect and coordinate the internal organs and surface tissues (19). During acupuncture treatment, needles are inserted into specific areas of the patient’s body called acupoints, which produces the sensations of soreness, numbness, fullness, or heaviness. These sensations are termed De Qi or Qi arriving, the process of which can regulate and re-balance the flow of Qi and can cure various diseases (20). A large number of studies have reported the efficacy and mechanism of acupuncture in the treatment of male infertility. This article describes acupuncture in treating male infertility from the efficacy to mechanism to provide an evidence-based decision about whether to use acupuncture in the treatment of male infertility.

## Search strategies

In the present study, the literature including clinical trials, animal experiment, meta-analysis, and systematic reviews from January 1985 and January 2022 were retrieved from the database of PubMed and CNKI. The search strategy included “male infertility”, “asthenozoospermia”, “oligoasthenozoospermia”, “varicocele”, “erectile dysfunction”, “premature ejaculation”, “genital inflammation”, “acupuncture”, “electroacupuncture”, and “transcutaneous acupoint electrical stimulation”.

## Causes of male infertility

**Figure 1:**
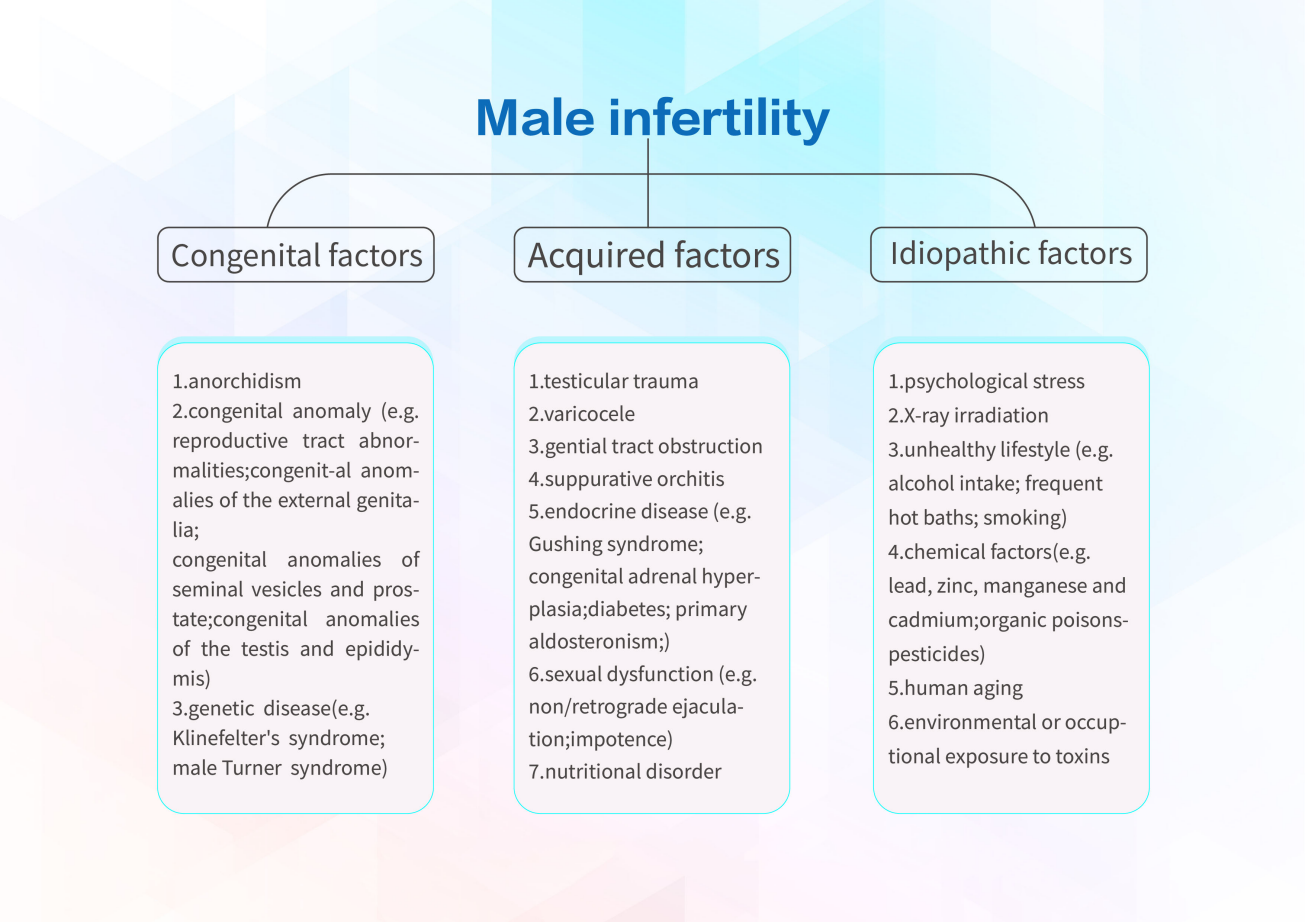
The causes and potential risks of male infertility.

There are many causes of male infertility, which can be mainly categorized into congenital factors, acquired factors, and idiopathic factors. The major congenital causes are divided into anorchia, congenital anomaly (e.g., reproductive tract abnormalities, congenital anomalies of the external genitalia, congenital anomalies of seminal vesicles and prostate, congenital anomalies of the testis, and epididymis), and genetic disease (e.g., Klinefelter’s syndrome, male Turner syndrome) (21). Congenital bilateral absence of the vas deferens is the main cause of infertility caused by reproductive tract abnormalities. It is well known as obstructive azoospermia, which is caused by obstruction of sperm transport during ejaculation, therefore absenting the spermatozoa in the ejaculate (22). Acquired factors include testicular trauma, varicocele, reproductive tract obstruction, suppurative orchitis, endocrine disorders (e.g., gusher syndrome, congenital adrenal hyperplasia, diabetes mellitus, and primary aldosteronism), sexual dysfunction (e.g. non-retrograde ejaculation and impotence), and nutritional disorders. The research conducted by Giorgio Cavallini et al. has found that sperm abnormalities (e.g., poor sperm quality, low sperm counts, and reduced sperm motility) account for nearly 30% of male infertility (23). In addition, nearly 50% of cases do not have a clear pathogenesis and refer to as “idiopathic infertility” (24). Age is one of idiopathic factors, which is the most non-collectable cause of male infertility. Testosterone levels and sperm concentration decrease with age, although some aging men can still be fertile by attempting multiple intercourse (25). In addition, several cases of infertility may potentially be induced by occupational exposure to toxic chemicals and lifestyle factors including tobacco smoking (26) and alcohol intake (27), and psychological stress can cause endocrine disruptions leading to male infertility. **Figure 1** shows all the causes and potential risks of male infertility.

## Acupuncture

TCM has been utilized to treat and prevent various diseases for thousands of years. Acupuncture originated about 3,000 years ago in China. In ancient China, it was found that stimulating specific areas of the body can relieve pain. Afterward, during the Neolithic period, people utilized sharpened stones and bones as appliances for drawing blood or lancing abscesses, which might be associated with the origin of acupuncture (28). In the following centuries, with the development of smelting technology, various types of needles came into being and were used to treat diseases. The Yellow Emperor’s Classic of Internal Medicine (Huang Dei Nei Jing), the first comprehensive and specific acupuncture system, mentioned that the needling sensation is essential in operation (19). Needling sensation, also known as obtaining qi, is the feeling when the needles are punctured into acupoints and manipulated, as the patients usually feel numbness, pain, soreness, etc. The curative effect is usually better in patients who have these sensations than those who do not (29). Guided by meridian and acupoint theory, acupuncturists regard human as entirety and employ acupuncture to treat and prevent diseases. TCM believes that meridians are a network of pathways in the human body that connect and coordinate with the internal organs and surface tissues (30). Diseases are usually caused by interruptions in the flow of “Qi” and “blood” in the meridians. Inserting needles or stimulating acupoints using acupressure can regulate Qi and blood and, therefore, can be used to treat diseases because, according to this theory, diseases are usually caused by interruptions of Qi and “blood” flow in the meridians. Stimulating acupoints by acupuncture needles or acupressure can regulate Qi and blood and can treat diseases.

Acupuncture has been used for more than 1,000 years worldwide. Japan was the first country to incorporate acupuncture into its medical system. In the seventeenth century, Christian missionaries brought acupuncture to Europe (31). In the nineteenth century, Louis Berlioz, a French doctor, first proposed the idea of electroacupuncture (EA), which is a modern treatment derived from traditional acupuncture, and by doing so, he has added electrical stimulation to the acupuncture science and increased the stimulation methods (32). Ultimately, Zhu Longyu invented the first EA instrument in the 1950s and applied it to the clinic. Numerous clinical studies have shown that the combination of needle and electric stimulation could augment the stimulus and enhance therapeutic effects on diseases (33, 34). Transcutaneous acupoint electrical stimulation (TAES) is another extension of acupuncture, in which electrodes are placed on acupoints to stimulate them through electric current to exert analgesia or other therapeutic effects (35). In addition, acupoint injection, catgut embedding, auricular acupuncture, and laser acupuncture are also invented in acupuncture science.

Up to now, there are 103 countries around the world that approved the use of acupuncture, of which 29 have formulated corresponding laws or regulations, and even several countries have covered this treatment in their medical insurance system (36). It is an invasive technique with ultra-micro-trauma and is usually regarded as low risk if operated by experienced personnel (37). In addition, the adverse effects of acupuncture were substantially lower than those of drugs or other conventional medical procedures under identical conditions (38). The World Health Organization has announced the range of conditions treated by acupuncture in the respiratory, circulatory, alimentary, urinary, endocrine, nervous, cardio-cerebral, reproductive systems, etc. (39–41). Acupuncture treatment of male infertility has been recorded in many ancient classics. In recent years, a growing number of clinical and animal studies have been conducted to study the treatment of male infertility with acupuncture to observe its efficacy and explore the mechanism.

## The overview of acupuncture in the treatment of male infertility

In China, the treatment of acupuncture for male infertility has a long history, which can be traced back to the classic *A–B Classic of Acupuncture and Moxibustion (Zhen Jiu Jia Yi Jing)* in the Western Jin Dynasty, nearly 2,000 years ago. The earliest study of acupuncture for male infertility in Western countries was conducted by Riegler et al. in 1984. They investigated the correlation between the effect of acupuncture on male fertility and psychological effects (42). The efficacy of treatment was evaluated by subjective parameters, namely, written psychological tests, and objective parameters, including concentration, volume, and motility of sperm before and after acupuncture. The result showed that apart from volume, the other sperm parameters increased significantly. Due to the fact that psychological test showed no change, it was suggested that the effect of acupuncture on improving sperm quality is not triggered by placebo mechanisms. This is the first report on the effectivity of acupuncture in treating male infertility in Western countries. In the following years, many countries around the world carried out studies on acupuncture treatment of male infertility and have reached similar conclusions (43–47). It was not until 2009 that Stefan Dieterle et al. designed the first prospective, randomized, single-blind, placebo-controlled trial of acupuncture for infertile patients with severe oligoasthenozoospermia based on the principles of TCM (48). A total of 57 patients were randomly assigned to receive either acupuncture or placebo acupuncture twice weekly for 6 weeks totally. The results showed that the percentage of motile sperm in the acupuncture arm is significantly higher than that in the placebo arm, which supports the significance of acupuncture in men with severe oligoasthenozoospermia.

Many scholars in the world have carried out in-depth study on acupuncture and created many new acupuncture therapies, such as the ones combining modern science and technology with traditional acupuncture that have been widely used. Cakmak et al. (49) has performed a prospective, randomized study to clarify the effect of the short-term EA on testicular blood flow (TBF) in men. This is the first study to show a point- and frequency-specific effects of EA on TBF, namely, a significant increase in TBF at 10 Hz within 5 min upon stimulation of Guilai (ST-29). More recently, a clinical trial on semen parameters in infertile patients with oligospermia has demonstrated that compared with the sham laser acupuncture group, the motility and concentration of sperm had a significant increase in the laser acupuncture group (p=0.0001) (50). In addition, since the laser probe is only in contact with the body surface, it is minimally invasive; therefore, it is safer, more convenient, and better tolerated by patients. A study evaluating the effect of transcutaneous electrical acupuncture point stimulation (TEAS) on sperm parameters found that 2 Hz TEAS can improve sperm count and motility in patients with abnormal semen parameters (51). With the increasing number of male infertility patients, there are more and more studies on acupuncture treatment of male infertility. In 2015, some scholars from Chengdu University of Traditional Chinese Medicine and Gansu University of Chinese Medicine in China searched and analyzed Chinese literature on acupuncture for male infertility (52). By analyzing the included 94 studies, they noticed that a total of 69 acupoints can be needled to treat male infertility, including 3 extra-meridian acupoints, 18 auricular acupoints, and 3 scalp acupoints. Among them, there are 15 acupoints with a frequency of more than 10 times, and CV4 (Guanyuan), BL23 (Shenshu), DU4 (Mingmen), SP6 (Sanyinjiao), KI3 (Taixi), and ST36 (Zusanli) are used as the basic acupoints for the clinical treatment of male infertility (the location of these acupoints are shown in **Figure 1**). Furthermore, some acupoints can be added or subtracted according to different TCM patterns. Meanwhile, several meta-analyses on whether acupuncture performed to improve male fertility was effective and safe have been published. Among them, the one conducted by Fang You et al. (53) has systematically assessed the effect of acupuncture on men with oligoasthenozoospermia. Based on the included 12 randomized controlled trials with 1,088 participants, acupuncture or acupuncture together with another intervention was effective in improving the semen quality. The efficacy of acupuncture on the treatment of male infertility is introduced further as follows.

## Clinical effect and mechanism of acupuncture on male infertility

### Semen parameters

Semen quality is usually considered to be a surrogate measure of fecundity in male fertility and pregnancy assessments. Abnormal semen parameters were found in approximately 50% of men evaluated for infertility (54). A comprehensive meta-regression analysis report demonstrated that during the past half a century, the number of sperm and other sperm parameters have shown a significant decline trend (55). Apart from oligoasthenospermia, other diseases that cause male infertility due to semen quality include oligospermia, asthenozoospermia, and teratozoospermia (56). In 1997, Siterman et al. (43) conducted a prospective controlled study to evaluate the effect of acupuncture on men who suffered from subfertility due to sperm impairment. After the treatment, all semen parameters of the 16 acupuncture-treated subfertile patient semen parameters such as total functional sperm fraction, percentage of viability, total motile spermatozoa per ejaculate, and integrity of the axonema were increased significantly compared with control group (p ≤ 0.05). Jin ZiRun et al. (57) investigated the effect of TEAS in the treatment of asthenozoospermia by needling acupoints BL23 bilaterally, left ST36, and CV4 once a day for 60 days. Compared with the sperm motility and vitality in baseline, TEAS treatment, either 2 or 100 Hz, significantly increased sperm motility and the percentage of grade a+b sperm in the asthenozoospermic patients. A Macedonian young infertile couple received acupuncture treatment in Skopje, Macedonia. After the treatment, the sperm quality of the man and ovarian function of the woman were restored, and their endocrine system was balanced (58). A prospective, controlled, and blind study was conducted by Gurfinkel, Edson et al. (47) to assess the effect of acupuncture and moxibustion on the semen quality in patients with semen abnormalities. After the treatment, compared with the control arm who received acupuncture and moxibustion at non-therapeutic acupoints, the ratio of the normal form sperm increased significantly in the treatment group.

The endocrine system is the main regulator of female and male reproductive function (59). The hypothalamic–pituitary-gonadal axis (HPGA), the regulatory center of reproductive endocrine activities, plays a role in maintaining normal reproductive function. The gonadotropin-releasing hormone (GnRH) secreted by the hypothalamus promotes the secretion of follicle stimulating hormone (FSH) and luteinizing hormone (LH) in the anterior pituitary. In men, FSH stimulates the Sertoli cells (SCs) to produce androgen binding protein (ABP), which formats the barrier of the blood–testis (60). FSH could increase the number of SCs and promote spermatogonial maturation by action on SC receptors. LH could act on interstitial Leydig cell, which is in synergism with the action of FSH on SCs (61), to aid in steroidogenesis and production of testosterone, thereby taking part in the appropriate regulation of spermatogenesis and consistency in semen quality. The abnormal level of FSH and LH would decrease testosterone synthesis. Testosterone, an androgenic steroid, is essential for the continuation of the normal spermatogenesis cycle and plays an integral role in spermatogenesis, sperm maturation, and sperm release. Whatever the reason, low levels of testosterone in the testes could reduce sperm production, leading to reduced fertility and even infertility.

The increased level of FSH and LH in low-fertility men are related to the damage of spermatogenic epithelium and stromal cells in testicular seminiferous tubules (62). A study conducted by Liu et al. found that EA can effectively reduce FSH and LH levels of adenine-induced spermatogenesis dysfunction model rats (63). Another study has shown that needling “Zhibian (BL54) through Shuidao (ST28)” and acupuncture on BL23 for 28 days can significantly reduce the level of LH in rats (64). Interestingly, other studies have also reached similar conclusion that acupuncture could significantly reduce the level of FSH and LH in patients with oligoasthenospermia (65, 66), which suggests that acupuncture may help reduce the damage of testicular seminiferous epithelium and mesenchymal cells caused by abnormal levels of FSH and LH, thereby improving male fertility. Acupuncture can also increase androgen levels in infertile men. Zeng et al. carried out a study on the effects of EA on cytochrome P450 side-chain lyase (P450scc) and steroid growth factor-1 (SF-1) in the testis of middle-aged and elderly rats with partial androgen deficiency syndrome (PADAM). The results showed that acupuncture on BL23 and CV4 could significantly increase the level of total testosterone and free testosterone in PADAM rats and improve the pathological manifestations of Leydig cells and SCs in the testis (67). Furthermore, Ren et al. also reached the same conclusion (68). Compared with PADAM rats, the levels of serum TT and FT were significantly increased; the protein and mRNA expression of P450scc, 17β-hydrox-ysteroid dehydrogenase 3 (17β-HSD3) and SF-1 were significantly increased in PADAM+EA rats. Therefore, the mechanism of acupuncture regulating androgen expression may be related to P450scc, SF-1, and 17β-HSD3.

It has been indicated that the poor sperm parameters are related to the changes in calcium and integrin-binding protein 1 (CIB1) and cell cycle regulator cyclin-dependent kinase 1 (CDK1). The imbalance of CDK1 or other cell cycle regulators can interfere with the normal interval of SC proliferation, resulting in the disproportion between SCs and germ cells, which increases germ cell apoptosis and leads to spermatogenesis defects (69). A clinical experimental study conducted by Yu Yan et al. has suggested that after treatments of TEAS 30 min daily for 30 days, the sperm count and motility in patients with abnormal semen parameters have improved, which is associated with the increased seminal plasma zinc, NAG, and fructose and the upregulation of CIB1 and downregulation of CDK1 (51). SCs, responsible for endocrine and paracrine duties, provide physical and nutritional support to germ cells during spermatogenesis (70). However, both physical and chemical alterations of SCs’ cytoskeleton could severely influence spermatogenesis (71). It has been indicated that after 10 sessions of EA, the sperm count, weight, sperm concentration, and sperm motility have increased. These improvements were connected with disrupted expression of cytoskeletal proteins of SCs and enhanced proliferation of germ cells with reduced apoptosis of germ cells (72). Its mechanism that EA enhances germ cell proliferation by improving the function of SCs facilitates the recovery of spermatogenesis and normal semen parameters in subfertile men (73). The research carried out by Jin ZiRun et al. found a novel mechanism for the pathogenesis of asthenozoospermia. The downregulation of CatSper channel in the sperm might be a contributor or a downstream indicator for asthenozoospermia, while 2 Hz TEAS or EA can be beneficial to recover through inducing the functional upregulation of CatSper channels in the sperm (74).

### Varicocele

The debate that varicocele causes male infertility has been going on for more than half a century. Male infertility caused by varicocele is the most easily cured, and even several patients are capable of having children without intervention (75). Varicocele, a kind of vascular disease, is manifested by abnormal dilatation and tortuosity of the pampiniform plexus veins, which can be observed in 35%–44% among men with primary infertility and 45%–81% with secondary infertility (75). Increasing lines of evidence have suggested that varicocele-mediated infertility is not triggered by a factor alone but is the result of the synergy of multiple factors, such as oxidative stress, hypoxia, and nutrient deprivation (76). At present, a large number of studies show that acupuncture can effectively treat male infertility mediated by varicocele. Kucuk et al. (77) conducted a study to compare the effect of the acupuncture with subinguinal microscopic varicocelectomy on sperm parameters and pregnancy rates in patients with primary infertility. They found that the efficacy of acupuncture seems to be equivalent to varicocelectomy in the primary infertile patients with semen abnormalities. Compared to the varicocelectomy arm, the increased sperm concentration was higher in the acupuncture arm (p = 0.039), and the pregnancy rate was the same in both arms (33%). Subsequently, Chen Dong et al. (78) evaluated the curative effect by observing the rheological indices of infertile patients with varicocele after acupuncture. One hundred nine cases in the treatment group received acupuncture, while the other 106 participants were administrated with drugs. After treatment, the diameter of varicocele in patients with grades I and II in the two groups was decrease (p<0.05), and the diameter of varicocele in patients with grade II in the treatment group was more significantly narrowed (p<0.01). Meanwhile, all sperm parameters were improved with longer fluidized time in the treatment group, which were better than those in the control group (p<0.05). A case report about the treatment of acupuncture in varicocele concluded that acupuncture could be an alternative therapy for varicocele (79). The patient received 10 sessions of treatments on acupoints including CV4, Taichong (LR3), ST36, SP6 with manual manipulation, and Zhongji (CV3), Qihai (CV6), and bilateral ST29 with EA; then, the symptom disappeared. The conclusion is acupuncture may be an effective alternative therapy for varicocele.

The exact mechanism of varicocele-induced male infertility is still not fully understood. It is a growing recognition that scrotal hyperthermia, a faulty venous valve causing the reflux of toxic metabolites, elevated hydrostatic pressure in testis, hypoxia, oxidative stress, and Leydig cell dysfunction are the proposed mechanisms for varicocele-induced male infertility (80). Both the overproduction of reactive oxygen species (ROS) and antioxidant deficiency can lead to oxidative stress. Studies have found that ROS levels including malondialdehyde (MDA) in seminal plasma of patients suffering from varicocele are increased, with a significant positive correlation with varicocele degree (81). Furthermore, varicocele is associated with the reduction in local and systemic antioxidant defense, including the reduction in antioxidant substances such as superoxide dismutase (SOD). Extensive research has shown that infertile men have lower levels of antioxidants in their semen (82). Current evidence shows that acupuncture can treat diseases by inhibiting oxidative stress. An animal experiment conducted by Zhang et al. (83) investigated the effect of acupuncture on the antioxidation function of the testis. After needling on CV4 and ST36, the activity of nitric oxide (NO), nitricoxide synthase, SOD, and catalase increased significantly, while the levels of lipid peroxide and MDA decreased markedly in the testis of elderly mice.

In addition, the reduced arterial blood flow of the testis and the reversed pressure gradient between the arterioles and venules might result in testicular hypoxia, thereby aging the testis and impairing spermatogenesis (84, 85). A prospective randomized study by Cakmak et al. (48) found that peak systolic velocity, volume flow, end-diastolic velocity, and the diameter and area of testicular artery were significantly improved after 10 Hz EA for ST29. Based on the results, its possible mechanisms were similarly mediated as a reflex response by the testicular sympathetic nerves controlled by supraspinal pathways. Yu et al. (86) reported a male adolescent patient with varicocele who was treated with an innovative acupuncture therapy, Fu subcutaneous acupuncture (FSN), and found that the patient’s testicular pain, abnormal dilatation, and tortuosity of the spermatic veins were significantly relieved, which might be through the mechanisms of improving relaxation of muscle compression and increasing local blood reperfusion to restore blood flow. Needle pricking therapy stimulates the sympathetic trunk and regulates the function of the gonadal axis and abdominal arteries and veins, which can significantly reduce the blood flow viscosity of varicocele-induced infertility, thereby increasing the testicular blood flow perfusion and oxygen supply and promoting blood circulation to eliminate varicocele (78, 87). In conclusion, acupuncture can enhance blood supply in the testicular artery, decrease the testicular temperature by heat exchange in the pampiniform plexus, and interrupt the perioxidation process, thereby providing a suitable environment for sperm survival, and then increase sperm concentration and improve fertility (88, 89).

### Reproductive system infection

Studies have found that 12%–35% of infertile men have had male reproductive system infection (90). Inflammatory damage to the testis, epididymis, and prostate can lead to changes in spermatogenesis, transport, and function. Clinically, many infertile men are usually complicated with prostatitis and epididymitis. Antibiotics are the mainstays of method in clinical practice; however, only one aspect could be relieved and seemed to have similar effects with placebo (91), and the efficacy tends to decline after drug withdrawal. In addition, the increased incidence of adverse events must be taken into consideration if used in the long term (92). Therefore, more and more patients utilize alternative therapies or antibiotics combined with acupuncture. In a study of fertility-related parameters in middle-aged men, normal sperm parameters were significantly reduced in patients with type II and III prostatitis, suggesting that prostatitis can lead to poor semen quality (93). Sun et al. (94) recruited a total of 440 men with prostatitis to receive 8-week acupuncture treatment. The results showed that acupuncture could significantly improve urination symptoms, pain, and sexual function in patients with prostatitis, with an effective rate of 60.6% (95%CI, 53.7%–67.1%). A study conducted by Siterman Shimon et al. (95) to verify whether acupuncture has an effect on the sperm output in patients suffering from inflammation of the genital tract with low sperm density. After treatment, the scrotal skin temperature and sperm concentration were improved, which suggested that acupuncture can be recommended for these men. Other studies have reached similar conclusions, but the reports lack indicators such as sperm motility related to male infertility. Larger randomized controlled clinical trials would be indispensable in the future to provide more convincing evidence for acupuncture in the treatment of male infertility caused by reproductive system infections.

Inflammation could raise the temperature of the testis/scrotum (96). Studies have found that a too high scrotal temperature can result in faster metabolism and increased oxygen consumption and impaired local microcirculation (95). The limited blood flow in the testes is insufficient to satisfy microcirculation, resulting in hypoxia and production of reactive oxygen species (97). The arterial blood flow of the testis decreases with each passing day in a body under oxidative stress. Pressure gradients between venous and arterial blood flow and impaired microcirculation may result in hypoxia of the testis. Testicular arterial blood flow reduction, hypoxia, and other pathological conditions may lead to testicular aging and impaired spermatogenesis. It has been proven in experimental research that the mechanism of acupuncture against aged rats with low testosterone is to reduce the inflammatory factors including NF-κB p65, COX-2, TNFα, and IL1β of Leydig cells, thereby promoting the synthesis of testosterone (98). It has been proven that acupuncture can produce anti-inflammatory effect by inhibiting the synthesis of cyclooxygenase in peripheral and central injurious sites (99). Apart from this, due to the systemic immunoregulatory actions of acupuncture, acupuncture itself might not eliminate pathogenic microorganisms but can be achieved by stimulating the body’s immune response (95, 100).

### Male sexual dysfunction

Erectile dysfunction (ED) and premature ejaculation (PE) are the two most common sexual dysfunction in men, which have affected 10%–52% of men in the world, as shown by epidemiological data (101). Sexual dysfunction usually occurs in men of reproductive age, and severe cases can lead to infertility (102). Now, more people seek complementary and alternative method for sexual dysfunction. The current evidence suggests that acupuncture might have beneficial effects on ED and PE. A prospective study conducted by Engelhardt et al. (103) investigated the efficacy of acupuncture in psychogenic erectile dysfunction (pED) patients. Twenty enrolled patients were randomized into the treatment group (acupuncture for ED) and placebo group (acupuncture against headache). After treatment, 68.4% participants in the treatment group received a satisfactory response, whereas 9% in the placebo group (p=0.0017), reaching the conclusion that acupuncture could be an effective option in patients with pED. Sunay, Didem et al. (104) carried out a study to determine the effectiveness of acupuncture in treating PE. Ninety patients were randomized into paroxetine, acupuncture, and sham acupuncture groups. After the treatment for twice a week for 4 weeks, they found the efficacy administrated by paroxetine was significantly better than that of acupuncture (p=0.001). However, the acupuncture had a significant stronger ejaculation-delaying effect than sham acupuncture. Studies have shown that EA can improve the symptoms of premature ejaculation by regulating the level of serum testosterone (105). Although most studies on acupuncture in the treatment of sexual dysfunction have some methodological problems including small sample sizes and lack of blindness, almost all current studies can prove the efficacy of acupuncture in the treatment of sexual dysfunction, at least better than placebo.

**Figure 2:**
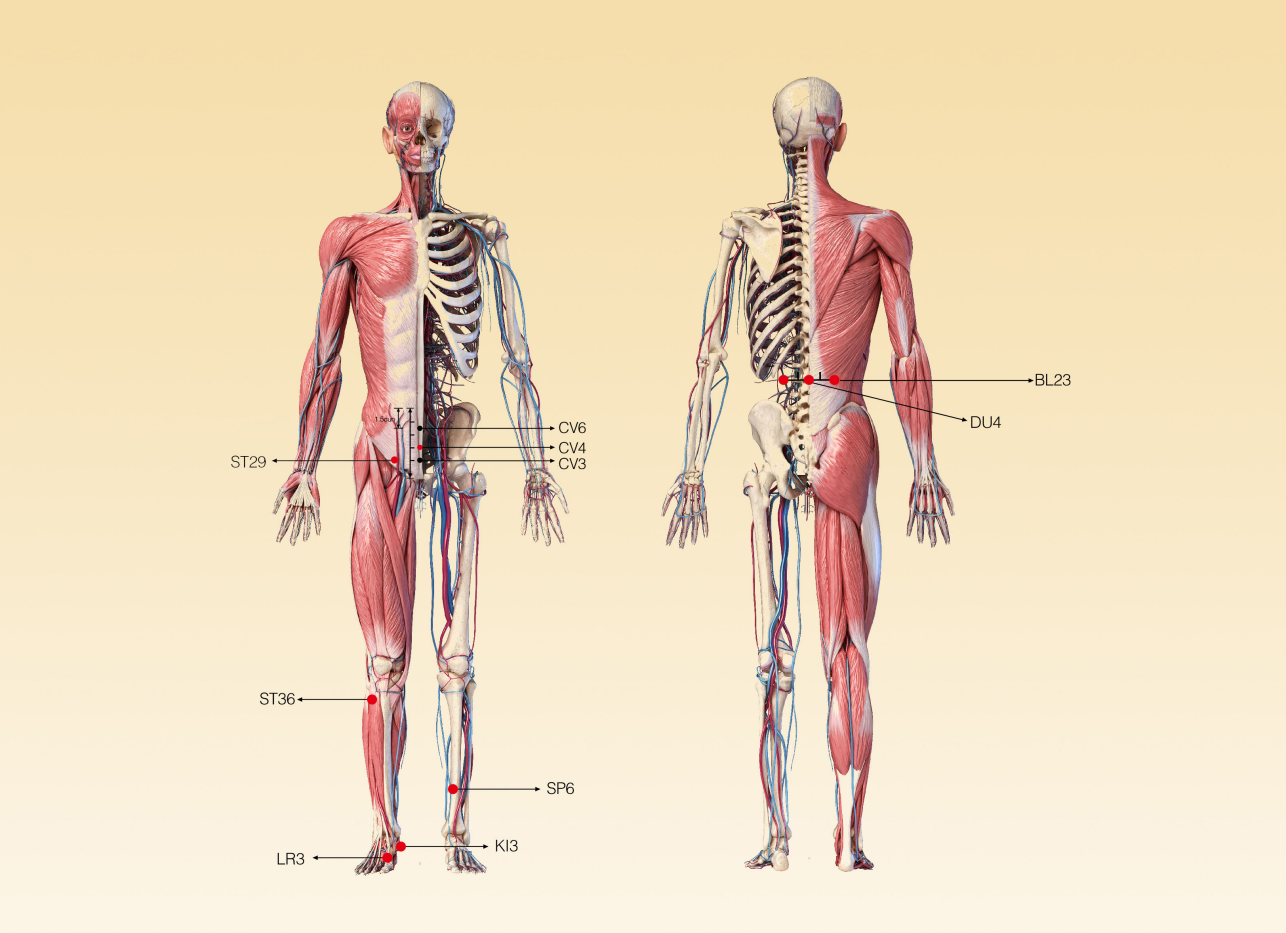
The location of the acupoints for the treatment of male infertility.

The initiation of penile erection is achieved by external stimulation of the somatic and autonomic nerves, and the perineal striated muscle is stimulated by sympathetic postganglionic fibers to maintain normal penile relaxation (106). The autonomic nerve contains sympathetic and parasympathetic fibers and originates from an important branch of the pelvic plexus, the cavernous nerve. The parasympathetic postganglionic fibers are distributed in the penile vascular smooth muscle, cavernous smooth muscle, and cavernous sinus small bundle column smooth muscle, and play a crucial role in the process of penile erection (107). The somatic nerves originating from the pudendal nerve transmit information to the spinal erection center located at the T12-L1 segment and the reflex erection center at S2–S4 to participate in reflex erection. Acupuncture on most acupoints located here can significantly increase the excitability of the spinal erection center, thereby improving the erection state (108). Therefore, its mechanism is related to the modification of sensory afferent impulses at the spinal level (109). For PE, it has been documented that acupuncture can treat PE through changes in central and peripheral neurotransmitters and neurohormones secretion and alter in blood flow regulation (110). It is also possible, although speculative, that acupuncture may alter the secretion of neurotransmitters such as selective serotonin-reuptake inhibitors (SSRIs), as shown in Sunay’s study (104). It has also been demonstrated that acupuncture promotes the release of serotonin, which plays a role in the central regulation of the ejaculatory reflex under the influence of several brain centers (111, 112). **Table 1** lists some of the studies mentioned above. The location, regional anatomy, and innervation of acupoints for male infertility mentioned above are described in **Table 2** and **Figure 2**.

**Table 1:** The studies of effective acupuncture for male infertility.

Study ID	Design	Samplesize	Number of treatments	Interventions	Outcomes	Limitation
40	Control study	32	10 sessions (twice a week for 5 weeks)	Treatment arm: Liegue (LU7),Zusanli(ST36),Pishu(BL20), Hegu(LI4),Quchi(LI11),Qichong(ST30), Xuehai(SP10),Fuliu(KI7),Neiguan(PC6), Yinlingguan(SP9),Ligou(LI5),Qihai(RE6), Shenmen(HT7),Zhaohai(KI6),Qugu(RE2), Sanyinjiao(SP6),Zhongliao(BL33), Huiyin (RE1),Guanyuan(RE4),Shenshu(BL23), Quguan(LI8), Mingmen(DU4) Control arm: no intervention	Compared with control arm, the total functional sperm fraction, percentage of viability, total motile spermatozoa per ejaculate, and integrity of the axonema in the intervention arm were improved (p < 0.05)	Small sample size No blindness
42	Control study	40	10 sessions (twice a week for 5 weeks)	Treatment arm: the main points: SP6, RE4, Zhaohai(KI6), LU7 and Qicong(ST30) “kidney-yang deficiency” syndrome add Taixi(KI3), Shenshu(BL23),Henggu(KI11) and Zhishi(BL52) “damp-heat in the genital system” syndrome add Shuidao(ST28), SP9, LI5, LI11 and Zuliqi(GB41) Control arm: no intervention	In the intervention arm: the sperm density from 0.3 ± 0.6 ×10^6^ to 3.3 ± 3.2×10^6^ spermatozoa per ejaculate; Z =−2.4, p ≤ 0.02); the sperm production from 0 to an average of l.5 ± 2.4×10^6^ spermatozoa per ejaculate (Z = −2.8, p ≤ 0.01)	Small sample size No blindness
43	Control study	22	8 sessions (twice a week for 2 months)	Baihui(GV20), PC6, Cice, SP10, Neiting (ST44), BL23, SP6, Xuanzhong(GB39), Gongsun(SP4), Fenglong(ST40), RE6 and RE4	Compared with before, acupuncture can improve sperm motility (11.0± 7.5%, p<0.01), normal sperm ratio (21.1%± 10.4% *vs* 16.2%± 8.2%, p<0.05) and the fertilization rates (66.2% *vs* 40.2%, p<0.01)	Small sample size Without control arm
44	Control study	19	20 sessions (twice a week for 10 weeks)	Treatment arm: ST30,KI3,Taichong(LR3),SP4,LI4,ST36, PC6,SP6,together with several moxa points Control arm:non-therapeutic points	Percentage of normal sperm in treatment arm presented a significant increase (calculated U=16.0, critical U=17.0) compared with control arm	Small sample size
45	RCT	57	12 sessions (twice weekly for 6 weeks)	Treatment arm: ST36, SP6, KI3, LV3, BL23, BL32, ST29, SP10, RE4, GV20 Control arm: Nonopenetrating placebo acupuncture needles	Acupuncture showed a significant effect of on the percentage of total motile sperm (p=0.035).	Small sample size
47	RCT	70	10 sessions (twice a week for consecutive 5weeks)	Treatment arm: laser fibers to HN1, BL19, ST36, SP9, SP6, LR3,RN12,RN6,RN4,BL18,LI4,RN2,BL21, BL20,Yanglingquan(GB34),Guilai(ST29),Tianshu(ST25),GB20,Dazhui(DU14),Dachangshu (BL25), and BL32 Control arm: sham laser acupuncture	The sperm motility and sperm concentration had a significant difference in both control and intervention group (p=0.0001)	Small sample size
48	RCT	121	60 sessions (once a day for 30 days, rest for 1–2days then continue with another 30 sessions	Arm 1: TEAS at 2Hz at BL23, ST36, CV1(Huiyin) and CV4 Arm 2: TEAS at 100Hz at BL23, ST36, CV1 and CV4 Placebo control arm: mock TEAS with a barely detectable current for 3s Control arm: given lifestyle advice	2 Hz TEAS can improve sperm count and motility in patients with abnormal semen parameters	Small sample size
51	Control study	72	60 sessions (once a day for 60 days)	2/100 Hz TEAS arm: bilateral BL23, ST36(left), RE4 Control arm: no intervention	Both 2 and 100 Hz TEAS are effective for the treatment of asthenozoospermia by improving sperm motility and vitality	Small sample size No blindness
52	Case report	1	12 sessions (once a week for 3 months)	HN1 (Sishencong), LI4, ST36, GB34, SP9, SP6, LR3, RN13 (Zhongwan), RN7, RN4, RN2 (Qugu), ST29, ST25, Fengchi(GB20), DU14,Ganshu(BL18),Danshu(BL19),BL20, BL21(Weishu),BL25 and Ciliao(BL32)	Acupuncture can improve the sperm quality in restoring fertility	Small sample size No blindness
55	RCT	90	8 sessions (twice a week for 4 weeks)	Paroxetine Acupuncture arm: ST36, LI4, KI3, LR3, Yintang(EX-HN3) and Zhongji(CV3) Placebo acupuncture	The effect of acupuncture was less effective than paroxetine, but was superior than placebo.	
56	Control study	50	24 sessions (rest for 1 day after 6 days sessions, totally4 courses)	The observation arm: 2-100 Hz EA on CV3 and SP6 The control arm: Longdan Xiegan decoction	EA was beneficial to premature ejaculation, which may be related to the regulation of serum testosterone.	Small sample size No blindness
58	Control study	30	16 sessions (twice a week for 8 weeks)	Acupuncture arm: CV3, CV4, CV6, BL23, BL32, and ST29 Varicocelectomy arm	Acupuncture is effective and similar with varicocelectomy in varicocele patients.	Small sample size No blindness
60	Case report	1	10 sessions	CV3,CV4,CV6, ST29,LI4, LR3, ST36, SP6, with EA at CV6 CV3 and ST29	Acupuncture may be an effective alternative therapy for varicocele	Small sample size No blindness

**Table 2:** The location, regional anatomy, and innervation of basic acupoints for male infertility.

Acupoint	Code	Location	Muscle	Innervation
Guanyuan	CV4	3 cun below the center of the umbilicus on the lower abdomen and on the anterior midline	Fibrous tissue, linea alba	L1
Shenshu	BL23	Under the 2nd spinous process of lumbar vertebra, next to 1.5cun	Erector spinae	L1
Mingmen	DU4	Below spinous process of 2nd lumbar vertebra	Erector spinae	L2
Sanyinjiao	SP6	3 cun proximal to the medial malleolus	Mm. flexor digitorum longus, tibialis posterior	L4–5, S1–2
Taixi	KI3	In depression between tip of medial malleolus and tendocalcaneous	Achilles tendon and plantaris tendon flexor pollicis longus	S2–3
Zusanli	ST36	3 cun below ST35, one finger- breadth from the anterior crest of the tibia, (front ridge of tibia), between fibula and tibia	Anterior tibial muscle, Extensor digitorum longus	L4–5, S1–2

## The controversy on acupuncture in the treatment of male infertility

In fact, it is not difficult to see that there are many defects in current studies on acupuncture treatment of infertility or improvement of male fertility, such as non-randomized controlled trials, and small sample size, which result in insufficient convincing conclusions of such clinical trials. Some studies have reported that sham acupuncture, placebo acupuncture, etc. used in the control group in acupuncture clinical trials can also produce therapeutic effects (113, 114). There are also studies showing that acupuncture versus placebo acupuncture has no statistically significant effect on *in vitro* fertilization outcomes (115). In addition, a meta-analysis conducted by Wen Jia et al. (116) suggested that compared with medications or sham acupuncture, acupuncture alone has no clear superiority in improving sperm quality. Although acupuncture may have the similar effect to medications, the considerable clinical heterogeneity and the poor methodological quality among the included studies made the present evidence inadequate to draw a definite conclusion. One crucial matter must be mentioned, that is, in the theory of TCM, the selection of acupoints is usually based on the different TCM syndrome types of diseases depending on the symptoms and signs of patients. However, to facilitate understanding and interpretation, the treatment protocol of the clinical acupuncture trials should be a fixed one, which followed the revised Consolidated Standards for Reporting Trials (CONSORT) statement (117). When the effect of acupuncture is evaluated, the TCM theory would be ignored due to the consideration of randomized controlled trial (RCT). Therefore, this standardization might weaken the effect of acupuncture in individual patients (48). In the future, some larger sample size, prospective, double-blind, placebo-controlled randomized controlled trials are needed to provide more convincing evidence of acupuncture in the treatment of male infertility, or we can spare no effort to update the standards for reporting interventions in controlled trials of acupuncture.

## Safety of acupuncture in the treatment of male infertility

In most cases, acupuncture is relatively safe, with few adverse events let alone serious complications (118). Short-term adverse events such as faint, stuck needle, bending of the needle, needle breakage, and hematoma may occur during the treatment if unqualified acupuncturists operate improperly, which can be avoided by careful manipulation. There are few reports on the adverse events of male infertility treated by acupuncture. In an RCT, among all patients, only one patient in the intervention arm reported dizziness and was relieved within 24 h under non-treatments (50). However, in other studies, there were adverse reactions related to acupuncture treatment. An *in vitro* fertilization (IVF) RCT conducted by Caroline A. Smith et al. reported that among 848 participants, 152 women had mild discomfort or bruises (119). In a prospective observational study enrolled that 229,230 patients to evaluate the safety of acupuncture, 8.6% participants reported experiencing at least one adverse events and 2.2% required treatment (120). Briefly, adverse events do occur during the acupuncture treatment, but to a large extent, they are mostly minor compared with non-acupuncture-related interventions.

## Summary

It is estimated that infertility has affected approximately 8%–12% of couples during reproductive age around the world (121). A Global Burden of Disease Survey reported that the age-standardized prevalence of male infertility has increased by 0.291% per year over the past 20 years (122). The current clinical therapy has shortcomings such as unsatisfactory efficacy, high cost, and many adverse reactions. Acupuncture, an adjuvant therapy, has aroused the curiosity of many scholars because of its controversial efficacy and mechanism. The merits of acupuncture in treating male infertility mainly include the following: (1) the advantages of acupuncture for male infertility include no side effects, few adverse effects, and low cost; (2) the combination of acupuncture and conventional therapy can improve the efficacy of male infertility; and (3) from the perspective of TCM, acupuncture can regulate the body as a whole, thereby treating male infertility. Currently, there are still some limitations in the existing studies, such as the lack of rigorous methodology and small sample size. Taken together, the present study not only summarizes the efficacy and mechanism of acupuncture for male infertility but supplies several instructive acupoints for clinic application as well. More high-quality RCTs and basic experiments are needed to provide a more persuasive evidence for the clinical application of acupuncture and more specific mechanism in treating male infertility.

## Author contributions

YZ: funding acquisition. JF, HH and YW: conceptualization and writing—original draft preparation. XZ, XYZ, TZ and MZ: editing. XW and YZ: supervision. All authors contributed to the article and approved the version.

## Funding

This work was supported by the National Natural Science Foundation of China (grant number 82074259), the Project of Cultivation project of outstanding youth fund of Heilongjiang University of Chinese Medicine (grant number 2018jc02), and “Outstanding Young Academic Leaders” scientific research project of Heilongjiang University of Chinese Medicine to YZ.

## Conflict of interest

The authors declare that the research was conducted in the absence of any commercial or financial relationships that could be construed as a potential conflict of interest.

## Publisher’s note

All claims expressed in this article are solely those of the authors and do not necessarily represent those of their affiliated organizations, or those of the publisher, the editors and the reviewers. Any product that may be evaluated in this article, or claim that may be made by its manufacturer, is not guaranteed or endorsed by the publisher.
